# Phosphorylation regulates human T-cell leukemia virus type 1 Rex function

**DOI:** 10.1186/1742-4690-6-105

**Published:** 2009-11-17

**Authors:** Matthew Kesic, Rami Doueiri, Michael Ward, O John Semmes, Patrick L Green

**Affiliations:** 1 Center for Retrovirus Research, The Ohio State University, Columbus, OH 43210, USA; 2 Department of Veterinary Biosciences, The Ohio State University, Columbus, OH 43210, USA; 3 Department of Molecular Virology, Immunology, and Medical Genetics, The Ohio State University, Columbus, OH 43210, USA; 4 Comprehensive Cancer Center and Solove Research Institute, The Ohio State University, Columbus, OH 43210, USA; 5 Department of Microbiology and Molecular Cell Biology and Center for Biomedical Proteomics, Eastern Virginia Medical School, Norfolk, Virginia 235070, USA

## Abstract

**Background:**

Human T-cell leukemia virus type 1 (HTLV-1) is a pathogenic complex deltaretrovirus, which is the causative agent of adult T-cell leukemia/lymphoma (ATL) and HTLV-1-associated myelopathy/tropical spastic paraparesis. In addition to the structural and enzymatic viral gene products, HTLV-1 encodes the positive regulatory proteins Tax and Rex along with viral accessory proteins. Tax and Rex proteins orchestrate the timely expression of viral genes important in viral replication and cellular transformation. Rex is a nucleolar-localizing shuttling protein that acts post-transcriptionally by binding and facilitating the export of the unspliced and incompletely spliced viral mRNAs from the nucleus to the cytoplasm. HTLV-1 Rex (Rex-1) is a phosphoprotein and general protein kinase inhibition correlates with reduced function. Therefore, it has been proposed that Rex-1 function may be regulated through site-specific phosphorylation.

**Results:**

We conducted a phosphoryl mapping of Rex-1 over-expressed in transfected 293 T cells using a combination of affinity purification and liquid chromatography tandem mass spectrometry. We achieved 100% physical coverage of the Rex-1 polypeptide and identified five novel phosphorylation sites at Thr-22, Ser-36, Thr-37, Ser-97, and Ser-106. We also confirmed evidence of two previously identified residues, Ser-70 and Thr-174, but found no evidence of phosphorylation at Ser-177. The functional significance of these phosphorylation events was evaluated using a Rex reporter assay and site-directed mutational analysis. Our results indicate that phosphorylation at Ser-97 and Thr-174 is critical for Rex-1 function.

**Conclusion:**

We have mapped completely the site-specific phosphorylation of Rex-1 identifying a total of seven residues; Thr-22, Ser-36, Thr-37, Ser-70, Ser-97, Ser-106, and Thr-174. Overall, this work is the first to completely map the phosphorylation sites in Rex-1 and provides important insight into the regulation of Rex-1 function.

## Background

Human T-cell leukemia virus types 1-4 are related complex retroviruses that are members of the genus *Deltaretrovirus *[1]. HTLV-1 and HTLV-2 are the most prevalent worldwide, whereas HTLV-3 and HTLV-4 were discovered recently in a limited number of individuals in Africa [2-4]. Of the HTLV isolates, only HTLV-1 infection has been clearly linked to the development of adult T-cell leukemia/lymphoma (ATL), an aggressive CD4+ T-lymphocyte malignancy, and various lymphocyte-mediated inflammatory diseases such as HTLV-1-associated myelopathy/tropical spastic paraparesis (HAM/TSP) [5-7]. However, a few cases of atypical hairy cell leukemia or neurologic diseases have been associated with HTLV-2 infection [8-12]. Although the difference in pathology between HTLV-1 and HTLV-2 has yet to be elucidated, it likely results from differential activities of the regulatory and accessory proteins.

In addition to the typical structural and enzymatic retroviral genes *gag*, *pol*, and *env*, HTLV encodes two trans-regulatory genes, *tax *and *rex*, which are essential for efficient viral replication/transformation, as well as several accessory genes important for viral infection and persistence *in vivo *[1]. The viral oncoprotein Tax increases the rate of transcription from the viral promoter located in the 5' long terminal repeat (LTR) [13-15] and modulates the transcription and activity of numerous cellular genes involved in cell growth, cell cycle control, DNA repair, and cell differentiation [16-20]. The pleiotropic effects of Tax make it essential for efficient viral replication as well as cellular transformation and oncogenesis [21-23].

HTLV-1 Rex (Rex-1) is a nuclear-localizing and shuttling phosphoprotein that acts post-transcriptionally by preferentially binding, stabilizing, and selectively exporting the unspliced and incompletely spliced viral mRNAs from the nucleus to the cytoplasm, thus controlling expression of the structural and enzymatic proteins that are essential for production of viral progeny [24-26]. Therefore, it has been proposed that Rex-1 regulates the switch from the early latent phase to the late productive phase of HTLV infection. Rex-1 binds viral RNAs via a *cis*-acting RNA sequence termed the Rex-response element (RxRE), which is located in the R region of the viral LTR [27]. Mutational analysis of Rex-1 has identified several critical domains including an arginine-rich N-terminal sequence that functions as an RNA binding domain (RBD) that overlaps with a nuclear localization signal (NLS), a leucine-rich central core activation domain that contains a nuclear export signal (NES), two flanking Rex-Rex multimerization domains, and a C-terminal stability domain [28-37].

Phosphorylation is a well known reversible regulatory event that controls the activity/function of proteins in eukaryotic cells [38]. It has been demonstrated that both Rex-1 and Rex-2 are phosphoproteins, and that this modification is critical for their function [26,39-42]. One study investigating the possible relationship of Rex-1 function and phosphorylation showed that treatment of HTLV-1 infected cells with the protein kinase C inhibitor H-7 [1-(5-isoquinolinyl-sulfonyl)-2-methylpiperazine] specifically blocked cytoplasmic accumulation of Rex-dependent *gag-pol *mRNA [40]. The same group reported that Rex-1 is phosphorylated on Ser-70, Ser-177, and Thr-174, with Ser-70 phosphorylation being 12-*O*-tetradecanoyl-phorbol-13-acetate (TPA)-dependent [39]. However, a complete phosphorylation map and the identification of the key residues required for function have yet to be elucidated.

In this study, we combined liquid chromatography tandem mass spectrometry (LC-MS/MS) analysis [43] of affinity-purified Rex-1 protein in combination with substitution mutational analysis to identify and functionally characterize key phosphorylation sites. The LC-MS/MS analysis achieved 100% coverage of the Rex-1 sequence and revealed five novel phosphorylation sites. We also identified two specific amino acid phosphorylation events found to be critical for Rex-1 function (Ser-97 and Thr-174). Overall, this work highlights the importance of phosphorylation and how it regulates the biological properties of Rex-1, ultimately controlling the distribution of viral gene expression and productive viral replication.

## Results

### Functional Domains of HTLV-1 Rex

**Figure 1:**
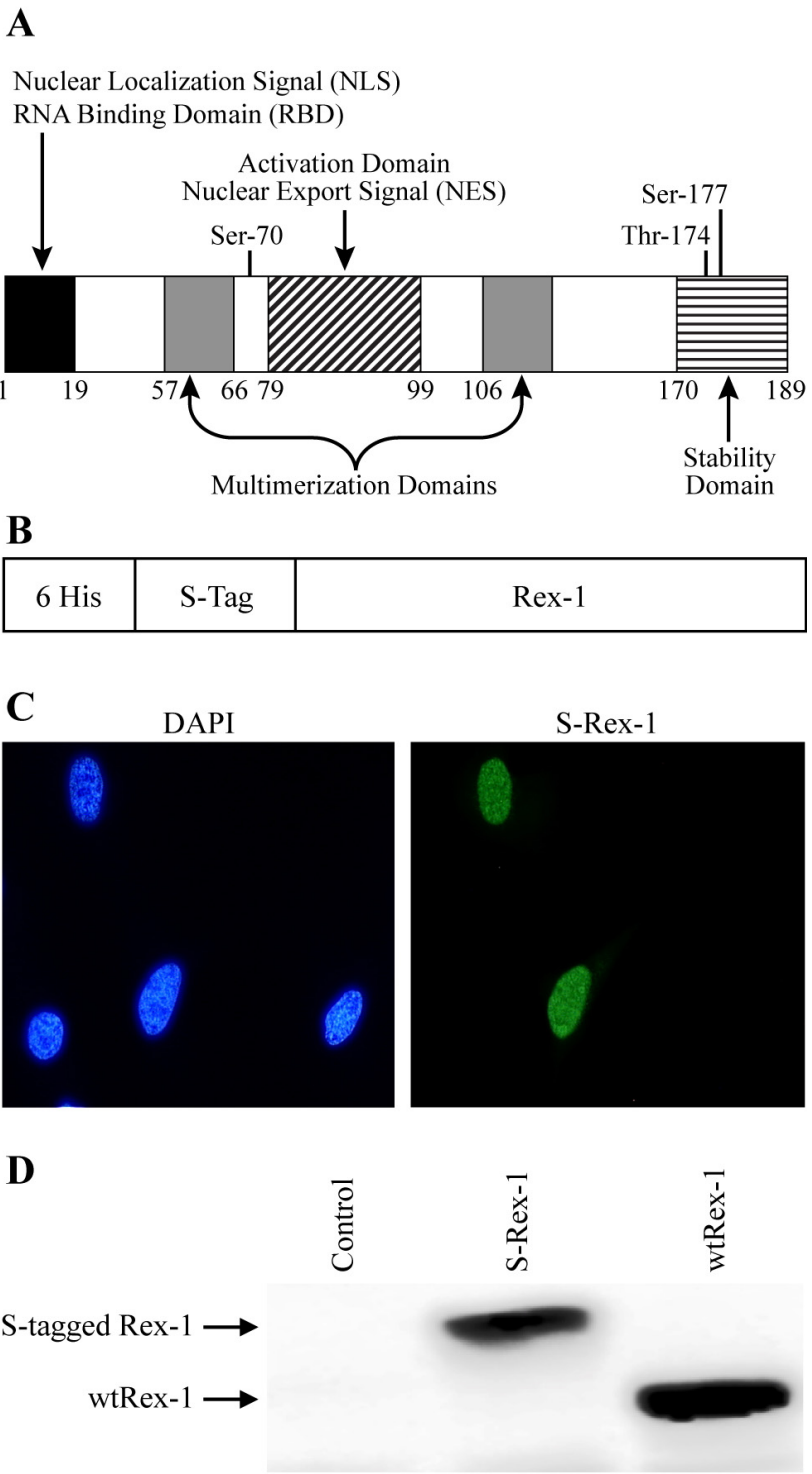
**Functional domains of HTLV-1 Rex and efficient expression and detection of affinity-tagged Rex-1**. (A) The functional domains of the 189 aa Rex-1 are depicted in shaded boxes. The nuclear localization signal (NLS) and the RNA binding domain (RBD) are positioned within the first 19 amino acids of the protein. The activation domain and the nuclear export signal (NES) are located between residues 79-99. This region is flanked by the two multimerization domains; the first lies between amino acids 57-66, whereas the second spans amino acids 106-124. Recently, a C-terminal stability domain was identified spanning amino acids 170-189 [28]. Three previously identified phosphorylation sites are indicated: Ser-70, Thr-174, and Ser-177 [39]. (B) Illustration of the S-tagged Rex-1 (S-Rex-1) expression vector construct (not drawn to scale). (C) To determine the subcellular localization of the S-tagged Rex-1, HeLa-Tat cells were transfected with 1 μg of S-Rex-1 or wtRex-1 expression plasmids. At 24 h post-transfection, cells were stained with rabbit α-Rex-1 specific antisera (Green). Nuclei were stained with DAPI (Blue). (D) Western blot of Rex-1 proteins expressed in 293T cells transiently transfected with S-Rex-1 and wtRex-1 cDNA plasmids. Proteins as indicated were detected using rabbit α-Rex-1 specific antisera.

Mutational analyses permitted the assignment of functional properties to distinct domains of the Rex-1 protein (Fig. 1A). In addition to the characterized nuclear localization signal/RNA binding domain, central core activation domain, two multimerization domains, and the newly identified C-terminal stability domain, three phosphorylation sites have been identified at Ser-70, Ser-177, and Thr-174 by the use of reverse-phase HPLC and sequential Edman degradation [39]. However, this approach only provided limited mapping coverage of Rex-1 and the functional relevance of the identified sites were not addressed. To date, no further studies have examined the possibility of other phosphorylation events or the effect of these post-translational modifications on Rex-1 function.

### Expression and Detection of Biological Active Affinity S-tagged Rex-1

**Figure 2:**
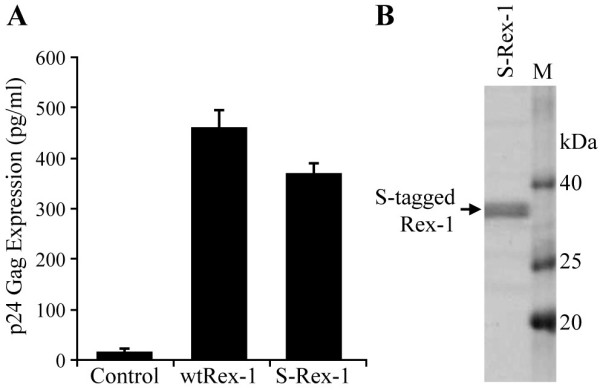
**Functional activity and expression of S-tagged Rex-1**. (A) The functional activity of S-tagged Rex-1 was determined using an HIV p24 Gag reporter assay. 293T cells were transfected with 0.25 μg pcTat, 0.5 μg pcGagRxRE-I, 0.05 μg CMV-luc, and 0.1 μg of wtRex-1 or S-Rex-1 expression plasmids. Twenty-four hours post-transfection, cells were harvested and assayed for p24 Gag. The values represent actual p24 Gag production from a representative experiment performed in triplicate. Error bars indicate standard deviations. (B) Affinity purification of S-tagged Rex-1 from mammalian cells. 293T cells were transfected with S-Rex-1 and S-tagged Rex-1 was purified by S-protein-agarose beads, eluted and resolved by SDS-PAGE analysis, and detected by Coomassie blue staining.

To identify the phosphorylated amino acid residues of Rex-1, we employed a tandem affinity purification approach of Rex-1 that was over expressed in mammalian cells. The S-tagged Rex-1 vector (S-Rex-1) expressed full-length Rex-1 protein fused to amino-terminal His_6 _and S-tags (Fig. 1B). Since the HTLV-1 regulatory proteins Tax and Rex are expressed from the same mRNA in partially overlapping reading frames, a point mutation was made in the nucleotide sequence that added a stop codon in the *tax-1 *reading frame that left the Rex-1 amino acid sequence unchanged [44]. This mutation completely abrogated Tax-1 protein expression and function (data not shown). The S-Rex-1 expression construct was transiently transfected into 293T cells, and the appropriate nuclear subcellular localization of Rex-1 was confirmed by indirect immunofluorescence microscopy (Fig. 1C). Wild type Rex-1 was shown as a single 27 kDa band by Western blot analysis using rabbit polyclonal α-Rex-1 antisera (Fig. 1D). Next we determined if the S-tagged Rex-1 retained its ability to function in our quantitative reporter bioassay in which HIV-1 p24 Gag production is measured and used as a read-out of Rex-1 functional activity in cultured cells. It is important to note that this assay has been a proven and accepted assay for Rex function and has been shown to directly correlate to Rex activity in the context of a molecular clone [25,28,45]. As shown in Figure 2A, S-Rex-1 displayed significant functional activity, although slightly lower than wtRex-1. We hypothesize that this reduced activity likely is due to the proximity of the amino terminal tag to the RNA binding domain. Taken together, these data demonstrate the proper nuclear subcellular localization and efficient expression of a functionally active S-tagged Rex-1 from mammalian cells.

### Affinity Purification of Rex-1 from Mammalian Cells

We successfully purified S-tagged Rex-1 protein from transfected 293T cells using S-protein-agarose beads as described in the "Methods". This purification procedure is based on the strong affinity between the 15-amino acid S-tag and the S-protein that is immobilized on the agarose beads, both of which are derived from RNase S [46]. The affinity purified S-tagged Rex-1 protein was resolved by SDS-PAGE and detected by staining with Coomassie blue (Fig. 2B). This purification process produced adequate quantities of highly purified S-tagged Rex-1 from mammalian cells and allowed the subsequent post-translational modification analysis by LC-MS/MS.

### Phosphopeptide Mapping of Rex-1 Using LC-MS/MS

**Figure 3:**
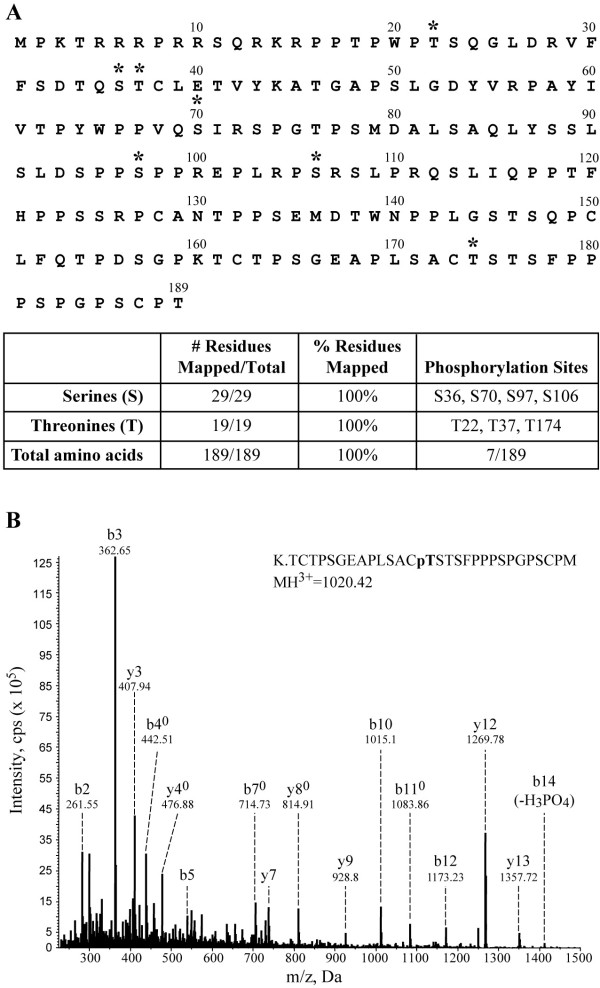
**Mapping Rex-1 phosphorylation sites by mass spectrometry**. (A) A compilation of the results obtained with LC-MS/MS analysis of S-tagged Rex-1. The 189 aa Rex-1 protein is depicted with phosphorylation sites identified (*). The table (inset) shows % total amino acid coverage from LC-MS/MS analysis. (B) A representative MS/MS spectrum of the tryptic phosphopeptide, which identified phosphorylation at Thr-174 is shown. CID Mass spectrum of m/z 1020.42 (3+) revealed a 29 aa peptide of M_r _3058.23. B and Y ion designations marked with (0) indicate a loss of H_2_O are doubly charged. The presence of the b10, b11 and b12 ions maps the phosphorylation to Thr-174. The MASCOT peptide score was 82 with an expected score of 0.0028.

Multiple strategies were employed to identify the phosphorylation sites within Rex-1. The affinity purified S-tagged Rex-1 band was excised and treated as follows. First, the protein was subjected to trypsin enzymatic digestion. The tryptic peptides that were too large to detect were either digested further with elastase or independently digested with elastase. This combined analytical approach allowed us to obtain a detailed physical map covering 100% of the Rex-1 sequence (Fig. 3A). Our analysis identified four serine phosphorylation sites at Ser-36, Ser-70, Ser-97, and Ser-106. We also identified three phosphorylated threonine residues at Thr-22, Thr-37, and Thr-174. Figure 3B shows a representative MS/MS spectrum of the tryptic phosphopeptide, which identified phosphorylation at Thr-174. We did not identify tyrosine site-specific phosphorylation, which is consistent with an earlier report [39].

### Substitutional Mutational Analysis of the Identified Rex-1 Phosphorylation Sites

**Figure 4:**
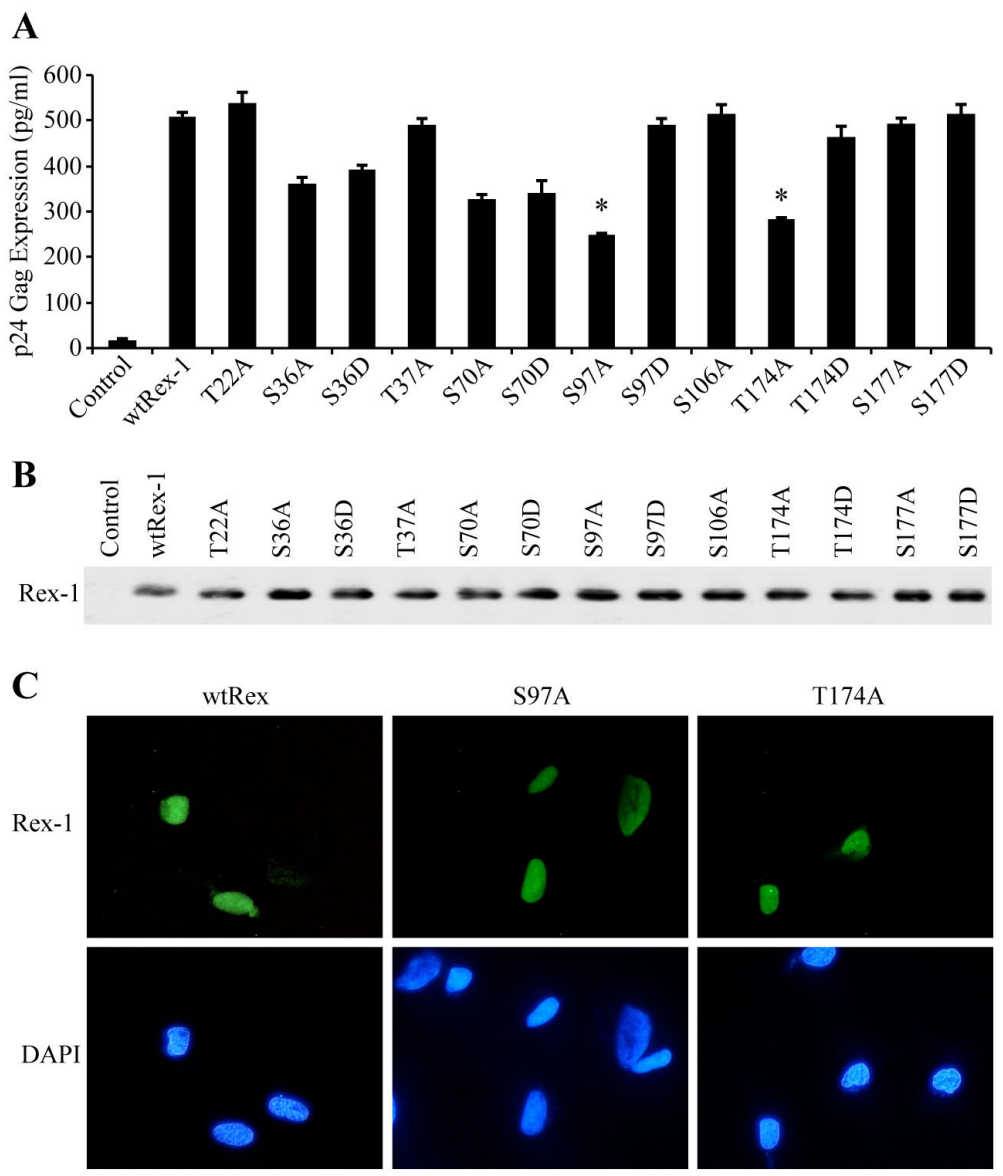
**Mutational analysis of Rex-1 phospho-specific mutants**. (A) The functional activity of either wtRex-1 or Rex-1 mutants, as indicated, was determined using the modified HIV p24 Gag reporter assay. The specific amino acid substitution for each Rex-1 mutant is shown. 293T cells were transfected with 0.25 μg pcTat, 0.5 μg pCgagRxRE-I, 0.05 μg CMV-luc, and 0.1 μg of wtRex-1 or Rex-1 mutant plasmids. At 24 h post-transfection, cells were harvested and assayed for p24 Gag. The values represent actual p24 Gag production from a representative experiment performed in triplicate. Error bars indicate standard deviations. T, threonine; S, serine; A, alanine; D, aspartic acid. (B) Western blot analysis of wild-type and Rex-1 mutants. Whole cell lysates normalized for transfection efficiency were subjected to Western blot using rabbit Rex-1-specific antisera. Rex-1 is indicated. (C) To determine the subcellular localization of the Rex-1 mutants, HeLa-Tat cells were transfected with 1 μg of a control plasmid, wtRex-1, or various Rex-1 mutants. At 24 hours post-transfection, cells were stained with rabbit Rex-1-specific antisera (Green). Nuclei were stained with DAPI (Blue).

To determine possible regulatory roles of the seven identified phosphorylation sites, we generated single alanine amino acid substitutions and tested these Rex-1 mutants to see if they retain their ability to function in our quantitative reporter bioassay. The Rex-1 mutants were transiently co-transfected into 293T cells with pcTat and pCgagRxRE-I, along with CMV-luciferase to account for transfection efficiency. We indentified two mutants S97A and T174A that displayed significantly reduced function (Fig. 4A). Further mutational analysis of these two residues by converting them to phosphomimetic aspartic acid (S97D and T174D) restored functional activity to wtRex-1 levels, which indicated that phosphorylation plays a positive functional role (Fig. 4A). Although we did not detect phosphorylation of Ser-177 in our analysis, we subjected this residue to a similar mutational and functional analysis. Our results indicated that mutant S177A or S177D maintained wild type Rex functional activity (Fig. 4A). Moreover, aspartic acid substitution of Thr-22, Ser-36, Thr-37, Ser-70, or Ser-106 had no effect on protein function, which is consistent with the conclusion that phosphorylation of any of these five residues does not negatively regulate function, but is silent (data not shown). The steady state expression levels of the wild-type and mutant Rex-1 proteins were determined for each mutant by Western blot analysis and detected using rabbit polyclonal α-Rex-1 antisera (Fig. 4B). All of the Rex-1 mutants were stably expressed. We previously showed that phosphorylation of a specific residue of Rex-2 at the carboxy terminus (Ser-151) is important for proper protein nuclear localization [28,33]. However, evaluation of the functionally disrupted substitution mutants S97A and T174A for subcellular localization revealed no difference when compared to wild-type Rex-1 (Fig. 4C). Together, we concluded that although phosphorylation of Ser-97 and Thr-174 are pivotal for Rex-1 function, the substitution for alanine did not result in a significant change in subcellular localization to the cytoplasm.

**Figure 5:**
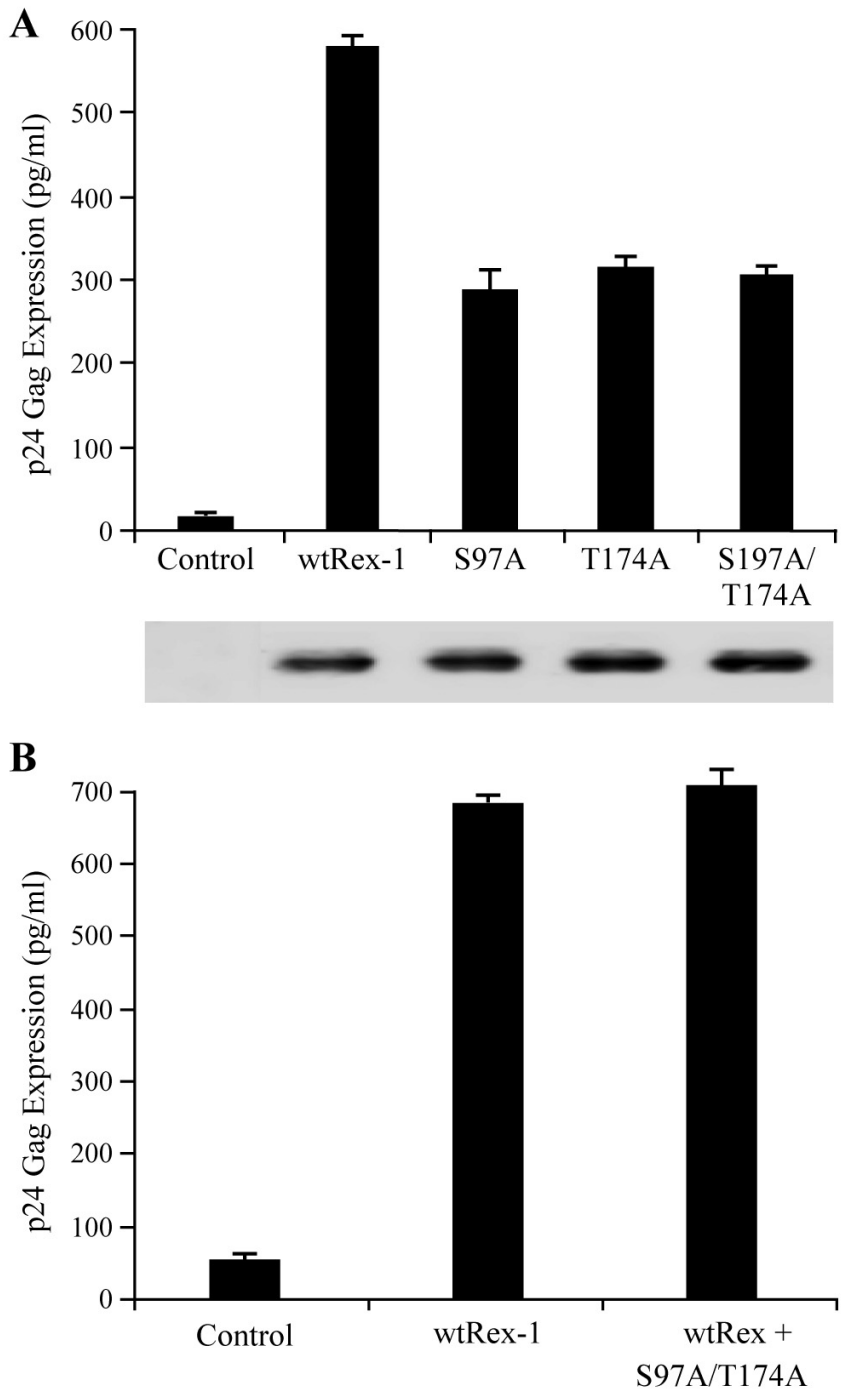
**The functional activity of S97A and T174A single and double mutants**. (A) The activity of either wtRex-1 or Rex-1 mutants, as indicated, was determined using the modified HIV p24 Gag reporter assay. The specific amino acid substitution for each Rex-1 mutant is shown. Cells were transfected and Rex activity was determined as described in the legend to Figure 4. The values represent actual p24 Gag production from a representative experiment performed in triplicate. Error bars indicate standard deviations. T, threonine; S, serine; A, alanine; D, aspartic acid. Whole cell lysates normalized for transfection efficiency were subjected to Western blot (shown below) using rabbit Rex-1-specific antisera. (B) 293T cells were transfected with 0.25 μg pcTat, 0.5 μg pCgagRxRE-I, 0.05 μg CMV-luc, and 0.1 μg of wtRex-1 or 0.1 μg of wtRex-1 + 0.2 μg of S97A, T174A Rex-1 mutant plasmid. Cell lysates were prepared 24 hours post-transfection and p24 Gag levels were determined by HIV-1 p24 Gag ELISA. Rex-1 functional assay reveals that the double mutant (S97A, T174A) does not inhibit the function of wtRex-1 and thus is not a *trans *dominant protein.

Since the individual mutations (S97A and T174A) still maintain partial function, it remained a possibility that phosphorylation of both residues are required for optimal biologic activity. To test this hypothesis and determine if there is a functional relationship between Ser-97 and Thr-174, we generated and characterized the double mutant for function and protein expression. As shown in Figure 5A, the double mutant S97A, T174A displayed significantly reduced functional activity as compared to wtRex-1, but a similar activity as the single mutants. Lastly, the nonfunctional Rex-1 mutants were next tested for their capacity to block the biological action of wtRex-1 using the pCgagRxRE-I reporter assay described above. The single mutants (S97A and T174A) or the double mutant (S97A, T174A) displayed a recessive negative phenotype, as the action of wtRex-1 was not significantly altered in their presence (Figure 5B and data not shown).

## Discussion

Phosphorylation plays a key role in regulating the function of cellular and viral proteins [28,38,39,47,48]. Previously, it was demonstrated that Rex-1 is a phosphoprotein and that phosphorylation may play a role in Rex-1 function [39,40]. It also has been shown that Rex-1 is essential for efficient viral replication and survival *in vivo *[45]. Given the importance of this protein in HTLV biology, we sought to understand how Rex-1 function is regulated. Multiple studies have been directed at understanding the importance of phosphorylation in HTLV Rex-2 function [26,33,41,42,49]. These studies reported that phosphorylation at the carboxy terminus of Rex-2 is critical for protein stability, shuttling, and cellular localization, all of which are positively regulated through phosphorylation [28,33,49]. There have been some efforts aimed at determining the role of phosphorylation in the regulation of HTLV Rex-1 [39,40]. The first studies used thin layer chromatography and tryptic peptide analysis. The studies reported that the native protein was phosphorylated mainly on serine and threonine. Subsequently, it was reported that Rex-1 was phosphorylated on three residues; Ser-70, Ser-177, and Thr-174. This group also speculated that protein kinase C may play a role in Rex-1 phosphorylation, which was supported by drug studies using the more global kinase inhibitor H-7 [40]. Neither study could conclusively identify all phosphorylation sites within Rex-1, nor were any of the sites further tested for their biological relevance.

In the current study, we were able to not only identify phosphorylated Rex-1, but also assign phosphorylation to site-specific residues by peptide sequencing using tandem mass spectrometry. Consistent with previous reports, we confirmed that Rex-1 is phosphorylated predominantly on serine and threonine residues. We report the identification of five novel phosphorylation sites, Thr-22, Ser-36, Thr-37, Ser-97, and Ser-106 and also confirmed the phosphorylation on Ser-70 and Thr-174. Furthermore, we identified specific phosphorylation sites that are critical for Rex-1 function *in vivo*. These phosphorylation sites specifically include Ser-97 and Thr-174. We previously showed that phosphorylation of a specific residue of Rex-2 at the carboxy terminus (Ser-151) is important for proper protein nuclear localization [28,33]. Evaluation of the functionally disrupted substitution mutants S97A and T174A for subcellular localization revealed no difference when compared to wild-type. It is important to note that Ser-97 falls within the previously characterized central core activation domain/nuclear export signal [50], and that phosphorylation of this residue may be pivotal for proper Rex-1 function. Previous studies of both HIV-1 Rev and HTLV-1 Rex showed that mutations within the NES interfere with the ability of these proteins to associate with CRM1, a cellular protein that belongs to the importin β family and functions as a nuclear export receptor for NES-containing proteins and the Rev- and Rex-dependent viral mRNAs encoded by these complex retroviruses [29,50-54]. An important direction for future studies is to evaluate whether the non-functional mutants are defective for CRM1 binding or the efficient interaction with the Rex-response element RNA target.

Thr-174, which is located in the carboxy terminus of Rex-1, was identified as a critical phosphorylation site. It was shown previously that Ser-151, located in the carboxy terminus of Rex-2, is a key phosphorylation site important for Rex-2 function *in vivo *[26,33]. We also demonstrated that Rex-1 and Rex-2 share a similar stability domain located within their carboxy terminus [28]. We hypothesized that phosphorylation of Thr-174 of Rex-1 (Fig. 4B) could play a similar role in regulating Rex-1 function similar to Rex-2 Ser-151. Further C-terminal comparison analysis is on-going to elucidate further homology between these two related proteins.

One previous study identified phosphorylation on Ser-177 of Rex-1 [39]. Throughout our studies, we were unable to confirm this finding, but we did identify multiple new sites. One explanation for why these new phosphorylation sites were not identified in the earlier studies could be that the high performance liquid chromatography fraction procedure used may have resulted in a loss of other phosphopeptides within the protein. The selective loss of phosphopeptides can result from the addition of a phosphate group, thus reducing hydrophobicity, which may cause failure of the protein to be retained on the reverse-phase material used in purification [55]. An additional consideration is that the previous study analyzed Rex-1 protein derived from a different cell type (COS-7 or HTLV-1 transformed T-cell lines), which may produce alternative post-translational modification patterns when compared to 293 T cells. Although it is not without its own caveat and limitations, LC-MS/MS provides a more robust method for the comprehensive mapping of phosphorylation sites [55-58].

## Conclusion

In summary, our data indicate that phosphorylation of specific residues regulates Rex-1 function. Utilizing a combination of affinity purification, liquid chromatography tandem mass spectrometry, and site-directed mutational analysis we identified two phosphorylated residues, Ser-97 and Thr-174 that are critical for Rex-1 function. Ongoing research in our lab is focused on comparative studies to better characterize the homology of the carboxy terminus of Rex-1 and Rex-2. These studies are focused on uncovering Rex cellular binding partners and kinase(s) and their functional relationship in order to better understand how phosphorylation regulates Rex-1 function. These studies will enable us to determine the differences between the two related proteins and perhaps gain insight into the distinct pathology following HTLV-1 and HTLV-2 infection.

## Methods

### Cells

293T and HeLa-Tat cells were maintained at 37°C in a humidified atmosphere of 5% CO_2 _in air in Dulbecco's modified Eagle medium. The medium was supplemented with 10% fetal bovine serum (FBS), 2 mM glutamine, penicillin (100 U/ml), and streptomycin (100 μg/ml).

### Mammalian Expression Plasmid

The Rex-1 expression vector SE356, which contains the HTLV-1 *tax/rex *cDNA expressed from the cytomegalovirus (CMV) immediate-early gene promoter, was described previously [14,59]. The S-tagged Rex-1 expression vector S-Rex-1 was constructed by inserting the HTLV-1 *tax/rex *open reading frame from SE356 into pTriEx4-Neo (Novagen, Madison, WI) in-frame with the amino-terminal His-tag and S-tag via SmaI and BamHI. All generated *rex *expression vectors contained a previously described mutation in the overlapping *tax *reading frame (F4Term), which had no effect on the Rex-1 amino acid sequence, but severely truncated Tax-1, completely knocking out expression and function [60]. The various *rex-1 *targeted mutations were generated using the QuikChange™ site-directed mutagenesis kit (Stratagene, La Jolla, CA) to introduce targeted amino acid changes. All mutations were confirmed by DNA sequence analysis and vector expression was verified by transfection and Western blot analysis. The human immunodeficiency virus type 1 (HIV-1) Tat expression vector, pcTat, Rex-1 reporter plasmid (pCgagRxRE-I) and the CMV-luciferase (firefly) transfection efficiency control were described previously [59].

### Rex-1 Functional Reporter Assay

The Rex-1 functional assay was performed as described previously with slight modification [26]. Briefly, 0.1 μg Rex-1 cDNA expression plasmid was co-transfected into 293T cells with 0.05 μg of CMV-luc, 0.25 μg of pcTat, and 0.5 μg of Rex-1 reporter plasmid pCgag-RxRE-I using Lipofectamine Reagent (Invitrogen, Carlsbad, CA). Cell lysates were prepared at 24 h post-transfection in Passive Lysis Buffer (Promega, Madison, WI) with a protease inhibitor mixture (Roche Applied Science Indianapolis, IN) on ice for 30 min. Luciferase activity was determined to control for transfection efficiency. HIV-1 p24 Gag levels in the cellular lysates were determined by ELISA (ZeptoMetrix, Buffalo, NY). All transfection experiments were performed in triplicate in three independent experiments and presented as an average with standard deviation.

### Immunoblot and Immunofluorescence Analysis

Cell lysates were prepared 24 h post-transfection in Passive Lysis Buffer (Promega, Madison, WI) with a protease inhibitor mixture (Roche Applied Science, Indianapolis, IN) on ice for 30 min. After centrifugation, total protein concentrations were determined by Bradford protein assay (Bio-Rad, Hercules, CA). To detect Rex-1, 50 μg of total cell lysates from transfected cells was separated by SDS-PAGE (12%) and transferred to a nitrocellulose membrane (Schleicher & Schuell Biosciences, Keene, NH). Proteins were visualized using polyclonal rabbit α-Rex-1 specific antisera and the enhanced chemiluminescence (ECL) Western blot analysis system (Santa Cruz Biotechnology, Santa Cruz, CA). Subcellular localization of Rex-1 was performed as previously described [61] with slight modification. HeLa-Tat cells were transfected with 1 μg of control plasmid or S-Rex-1. At 24 h post-transfection, cells were washed and fixed in PBS containing 2% paraformaldehyde and permeabilized in PBS containing 0.2% Triton X-100 and 0.5% FBS for 15 min at 4°C. Cells were incubated in blocking buffer (0.5% FBS and 2 mg/ml human IgG) for 30 min at room temperature. Staining was conducted in blocking buffer with rabbit α-Rex-1 specific antisera followed by secondary antibody conjugated to FITC Alexa 488 (Molecular Probes, Eugene, OR). Nuclear staining was performed using 4'6-diamidino-2-phenylindole (DAPI) Slow Fade Gold (Invitrogen, Carlsbad, CA). Fluorescence was visualized on an epifluorescence microscope (Olympus, Melville, NY) and digital images were taken using the Optronics Imaging System (Goleta, CA).

### Purification of Rex-1 Protein

Protein purification was performed as described previously with a slight modification [43]. Briefly, cell lysate (1.5 ml) was incubated with a 75 μl bed volume of S-protein agarose (Novagen) overnight at 4°C, washed twice with a high salt modified RIPA buffer (0.05 M Tris-HCl, pH 8.0, 0.1% SDS, 1% Triton X-100, 1.0 M NaCl, 0.01 M EDTA) and twice with a low salt modified RIPA buffer (0.05 M Tris-HCl, pH 8.0, 0.1% SDS, 1% Triton X-100, 150 mM NaCl). One hundred μl SDS loading dye with β-mercaptoethanol was added to the washed beads followed by boiling for 2 min. Samples were electrophoresed on a 12% SDS one-dimensional polyacrylamide gel and visualized by Coomassie blue staining. The S-tagged Rex-1 band was excised from the gel for further proteomic analysis.

### Mass Spectrometry Analysis

LC-MS/MS analysis was performed as described previously with slight modification [43]. Briefly, the S-tagged Rex-1 protein band was excised from a 1-D polyacrylamide gel, cut into 1-2 mm cubes, washed three times with 500 μl ultra-pure water and incubated in 100% acetonitrile for 45 min. Samples were reduced with 50 mM DTT at 56°C for 45 min and then alkylated with 55 mM iodoacetamide for 1 h at room temperature. The material was dried in a speed-vac, rehydrated in a 12.5 ng/μl modified sequencing grade trypsin solution (Promega, Madison, WI) and incubated in an ice bath for 40-45 min. The excess trypsin solution was removed and replaced with 40-50 μl of 50 mM ammonium bicarbonate, 10% acetonitrile (pH 8.0), and the mixture was incubated overnight at 37°C. Elastase digests were performed as described for trypsin at an enzyme concentration of 15 ng/μl, but were performed without acetonitrile in the reaction buffer. Peptides were extracted twice with 25 μl 50% acetonitrile, 5% formic acid and dried in a speed-vac. Digests were resuspended in 20 μl Buffer A (5% acetonitrile, 0.1% formic Acid, 0.005% heptafluorobutyric acid) and 3-6 μl were loaded onto a 12 cm × 0.075 mm fused silica capillary column packed with 5 μM diameter C-18 beads (The Nest Group, Southboro, MA) using an N2 pressure vessel at 1100 psi. Peptides were eluted over 55 min by applying a 0-80% linear gradient of Buffer B (95% acetonitrile, 0.1% formic acid, 0.005% HFBA) at a flow rate of 150 μl/min with a pre-column flow splitter resulting in a final flow rate of ~200 nl/min directly into the source. In some cases, the gradient was extended to 150 min to acquire more MS/MS spectra. An LTQ™ Linear Ion Trap (ThermoFinnigan, San Jose, CA) was run in an automated collection mode with an instrument method composed of a single segment and five data-dependent scan events with a full MS scan followed by four MS/MS scans of the highest intensity ions. Normalized collision energy was set at 35, activation Q was 0.250 with minimum full scan signal intensity at 1 × 10^5 ^with no minimum MS^2 ^intensity specified. Dynamic exclusion was turned on and utilized a three minute repeat count of 2 with the mass width set at 1.0 m/z. Sequence analysis was performed with TurboSEQUEST™ (ThermoFinnigan, San Jose, CA) or MASCOT (Matrix Sciences, London GB) using an indexed Human subset database of the non-redundant protein database from National Center for Biotechnology Information (NCBI) web site http://www.ncbi.nlm.nih.gov/.

## Competing interests

The authors declare that they have no competing interests.

## Authors' contributions

MK generated all the clones, carried out functional assays, purified the protein, and drafted the manuscript. RD characterized the Rex non functional mutants for trans-dominant activity and helped with editing the manuscript. MW performed the LC-MS/MS analysis and helped interpret the data. OJS helped in finalizing the manuscript and provided important input on the design of the protein expression and purification. PLG conceived the study, participated in its coordination, helped in drafting and finalizing the manuscript. All authors read and approved the final manuscript.
